# Neuropsychopharmacological effects of midazolam on the human brain

**DOI:** 10.1186/s40708-020-00116-y

**Published:** 2020-11-10

**Authors:** Junkai Wang, Pei Sun, Peipeng Liang

**Affiliations:** 1 School of Psychology, Capital Normal University, Haidian District, Beijing, 100048 China; 2 Beijing Key Laboratory of Learning and Cognition, Beijing, China; 3 Department of Psychology, Tsinghua University, Haidian District, Beijing, 100084 China

**Keywords:** Midazolam, Sedation, Anesthesia, Functional imaging, Consciousness

## Abstract

As a commonly used anesthetic agent, midazolam has the properties of water-soluble, rapid onset, and short duration of action. With the rapid development in the field of neuroimaging, numerous studies have investigated how midazolam acts on the human brain to induce the alteration of consciousness. However, the neural bases of midazolam-induced sedation or anesthesia remain beginning to be understood in detail. In this review, we summarize findings from neuroimaging studies that have used midazolam to study altered consciousness at different levels and content. We also compare the results to those of neuroimaging studies using diverse anesthetic agents and describe the common neural correlates of anesthetic-induced alteration of consciousness.

## Introduction

Midazolam is a widely used intravenous anesthetic agent with rapid onset, short duration of action and relatively rapid plasma clearance. It is mainly used to produce preoperative sedation and the induction of general anesthesia [1, 2]. Every year, millions of patients undergo anesthesia, drugs that induce an anesthetic state of diminished level of consciousness [3]. Despite the inestimable value to the medical and surgical services, the fundamental question of what is the neural mechanism by which anesthetic agents induce alteration of consciousness remains unanswered.

In the past decades, with the development of the field of neuroimaging, significant progress has been made in understanding functional changes in human brain associated with consciousness. Functional imaging methods are comprehensive, real-time, non-invasive techniques and show in vivo metabolic and functional changes. These methods, especially positron emission tomography (PET) and functional magnetic resonance imaging (fMRI), have been extensively used for exploring neural correlates of anesthetic-induced altered consciousness [4, 5]. As an experimental paradigm, anesthetic-induced anesthesia combined with functional neuroimaging presents a unique approach for studying neural responses as a function of consciousness [6].

This review first summarizes the pharmacology, pharmacodynamics of midazolam and altered cognitive function during midazolam-induced sedation. Then, we review findings from neuroimaging studies that have used midazolam to study changes in brain activity at reduced levels and content of consciousness, both with stimulation task and at resting state. In the following, we compare these results to those of neuroimaging studies using diverse anesthetic agents and describe the common neural correlates of anesthetic-induced alteration of consciousness.

## Structure, pharmacodynamics, and pharmacokinetics of midazolam

Midazolam is a 1,4-imidazole benzodiazepine and the first water-soluble benzodiazepine [1, 7]. Midazolam structure is unique and characterized by environmental pH-dependent ring-opening phenomenon. The drug is highly water-soluble at pH values of less than 4 and becomes much more lipid soluble at physiological pH [1, 7]. This characteristic contributes to rapid onset of action and minimizing pain on injection [1, 7].

Pharmacodynamics of midazolam is similar to those of other benzodiazepine drugs, including sedation, anxiolytic, anterograde amnesia, muscle relaxant, anticonvulsant effect, as well as hypnotic activities [1, 8–11]. Almost all these effects can be explained through its action on gamma­aminobutyric acid (GABA) receptors. GABA receptors mediate inhibitory neurotransmission in the central nervous system [12, 13]. Two separate receptors for GABA and benzodiazepine couple to an ion channel selective for chloride [14]. Midazolam binds to the benzodiazepine site on GABA-A receptors, the major GABA receptor, which enhances the effects of GABA by increasing the frequency of chloride channel opening leading to membrane hyperpolarization and neuronal inhibition [10].

Absorption of midazolam is rapid regardless of the administration route. After oral doses of midazolam, peak plasma concentrations generally occur between 0.5 and 1 h [15, 16]. In comparison with oral intake, midazolam has more rapid absorption after intramuscular injection or intravenous (IV) route, with peak serum concentrations as early as a few minutes [17]. For IV administration, the distribution half-life of midazolam has been recorded as 6 to 15 min. The elimination half­life is 1.7 to 2.4 h. The duration of action is 60 to 120 min [10, 18]. In addition, midazolam is extensively bound to plasma proteins with the binding occurring primarily in serum albumin. The lipophilic nature of midazolam accounts for the relatively rapid membrane penetration and fast onset of action [10]. Because of its rapid onset, short duration of action, water solubility, and relatively rapid plasma clearance, midazolam has proved to be one of the mainstays of sedation or anesthesia in clinical practice. Midazolam primarily undergoes metabolism in the liver and gut by the cytochrome P-450 CYP3A4 enzyme system and glucuronide conjugation [19].

Since midazolam is unique among benzodiazepines with rapid onset of action, good effectiveness and few adverse effects, it is one of the most commonly used sedative medications for a variety of therapeutic and diagnostic procedures [10]. Midazolam can be administered through oral, intravenous, intranasal, and intramuscular routes. Because of its water-soluble nature, it is well suited for continuous infusion when intravenous administration of other medications is not feasible [20]. The sedative actions of midazolam occur without loss of airway reflexes or significant adverse reactions in the autonomic, hormonal and circulatory systems, or causing nausea and vomiting. It is also useful for sedation in endoscopic procedures. Generally, the best use of midazolam in anesthesiology appears to be as an intravenous sedative agent for endoscopy [8–10] and regional anesthesia [1, 7].

## Altered cognitive function during midazolam-induced sedation

Anesthesia aims to enhance patient comfort, improve operating conditions (e.g., lack of coughing, muscular relaxation), and prevent recall of noxious physical and emotional experiences during the medical procedures. Midazolam has a great advantage in eliminating adverse experiences because of its marked effects on cognitive functions. As mentioned before, midazolam acts on GABA-A receptor to reduce the excitability of neurons, resulting in impairment of multi-domain cognitive functions [1, 12].

Midazolam, in common with other benzodiazepines, has strong anterograde amnesic property [11]. The anterograde amnesia limits the recollection of events after the drug is given and impairs ability to retain new information. The effect of midazolam on memory varies across its different stages and types [21, 22]. Memory processes a limitless amount of information, and information is stored in different forms like meaning, sounds and images. From storing information to the final recalling information, memory can be categorized into three stages: encoding, storage and retrieval [23]. Encoding is the first stage of memory. This stage accumulates the information from the surrounding and encodes it in a form that can be kept in memory. Storage and retention deal with the nature of memory where the information is stored, the time duration of the memory, and the amount of information that can be stored. Retrieval, the third process, refers to retrieving information out of our memory storage and back into conscious awareness [23]. Midazolam impairs the encoding of new information while having no deleterious effects upon either retention or retrieval of information acquired before drug administration [21, 22]. In addition, the longer the delay between acquisition and retrieval is, the greater the drug effect on performance is [21, 22].

**Fig. 1:**
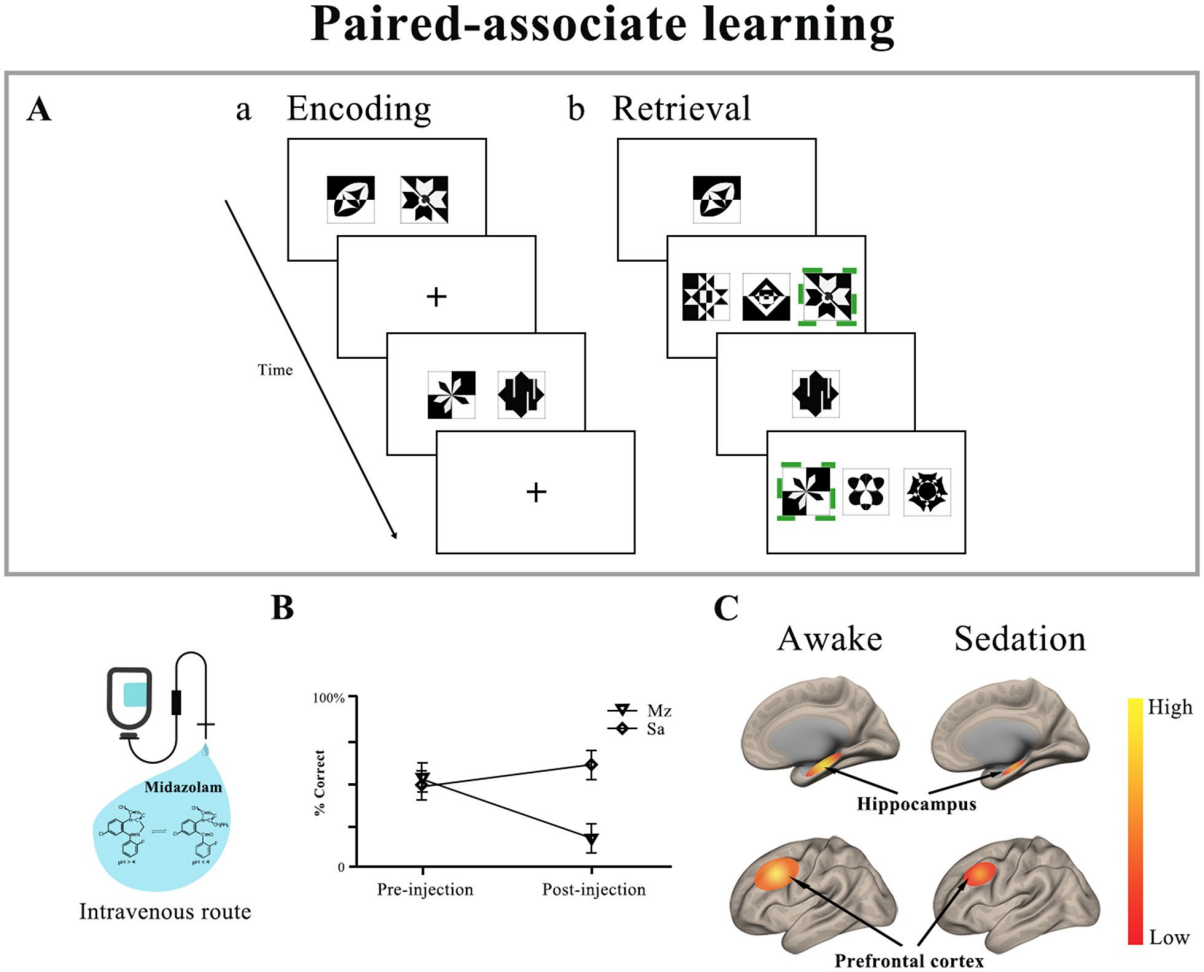
The effect of midazolam on visual paired-associate learning. **A** A schematic of the two parts of the visual paired-associate learning task consisting of (a) an encoding task, and (b) a cued recall task. **B** The effect of midazolam on encoding and retrieval of new information after drug administration. Individuals with intravenous midazolam showed a marked decrease in encoding and retrieval trials than subjects with saline. The hippocampus and prefrontal cortex were involved in paired-associate learning and midazolam significantly decreased the activation of hippocampus and prefrontal cortex in trials requiring encoding and retrieval of new information

For types of memory, one of the most popular concepts of memory suggests that there are three basic memory types: sensory memory, short-term memory, and long-term memory. Sensory memory refers to the initial process of storing information that is a very brief recall of a sensory experience, such as what we just saw or heard. Short-term memory, also refers to as primary memory, is that brief period of time where you can recall information you were just exposed to. Long-term memory, also calls secondary memory, encompasses memories that range from a few days to decades. In order to learn something successfully, information has to move from the sensory or the short-term memory to the long-term memory [24]. There are other definitions of memory. For example, long-term memory can be divided into two types of memory, explicit or conscious memory and implicit or unconscious memory [25]. In addition, explicit memory is subdivided into episodic and semantic memory [26]. There is no evidence that midazolam has effects on sensory memory and several studies suggest that the drug do not impair short-term memory [21, 22]. Sarasin et al. [27] performed a study and used digit symbol substitution tests to compare the effects of midazolam on explicit and implicit memory. The results showed that subjects who ingested midazolam performed significantly more poorly on the explicit memory task. Unlike explicit memory, implicit memory of midazolam group resisted impairment. Besides, while midazolam greatly impairs episodic memory, it appears not to affect semantic memory [21]. The highly sensitive memory tasks to the midazolam include serial learning and paired-associate learning [21] (Fig. 1).

Along with midazolam-induced anterograde amnesia, a previous study also suggested that subjects with intravenous midazolam showed a marked enhancement in implicit transitive inference (TI) performance [28]. Transitive inference is an ability to derive a relation “A is greater than C” from the premises “A is greater than B” and “B is greater than C”. Pigeons, fish, rats, chimpanzees, and humans have all shown capability of this [29–33]. TI task assesses the ability to generalize learned knowledge to new contexts [34]. Successful performance by animals and humans in implicit TI may be explained by reinforcement learning processes which depend on the striatal dopamine system [28, 34]. So, midazolam biases participants to recruit striatum during learning and this change actually supports the generalization of learned behavior to novel situations [28, 34].

## Cerebral blood flow during midazolam-induced sedation

In the past decades, with the development of the field of neuroimaging, significant progress has been made in understanding of the brain mechanisms associated with midazolam-induced sedation. Arterial spin labeling (ASL) methodology is a MRI technique used to assess cerebral blood flow (CBF) noninvasively by magnetically labeling inflowing blood [35]. In contrast to blood oxygen level-dependent (BOLD) signal, which is complex and depends on simultaneous changes in blood flow, vascular volume and oxygen metabolism, ASL perfusion imaging uses the arterial blood water protons as an endogenous contrast agent to direct measurement of tissue blood flow. In addition, ASL includes the lower inter-subject and inter-session variation, and is minimally affected by baseline drift, making it suitable for long-term studies with low-frequency changes [36, 37]. ASL perfusion technique is, therefore, especially suited to quantify physiological and psychopharmacological effects on the human brain. In one of our previous studies, ASL perfusion imaging technique was used to investigate the intrinsic CBF changes evoked by midazolam-induced light sedation. It was found that CBF in the bilateral medial thalamus and precuneus/posterior cingulate cortex (PCC) was specifically decreased for midazolam administration [38]. In line with this study, at similar depths of sedation, one recent study investigated resting-state CBF changes during mild sedation with propofol and demonstrated propofol-induced suppression of key cortical and subcortical regions including the bilateral paracingulate cortex, premotor cortex, Broca’s areas, right superior frontal gyrus and also the thalamus [39].

**Fig. 2:**
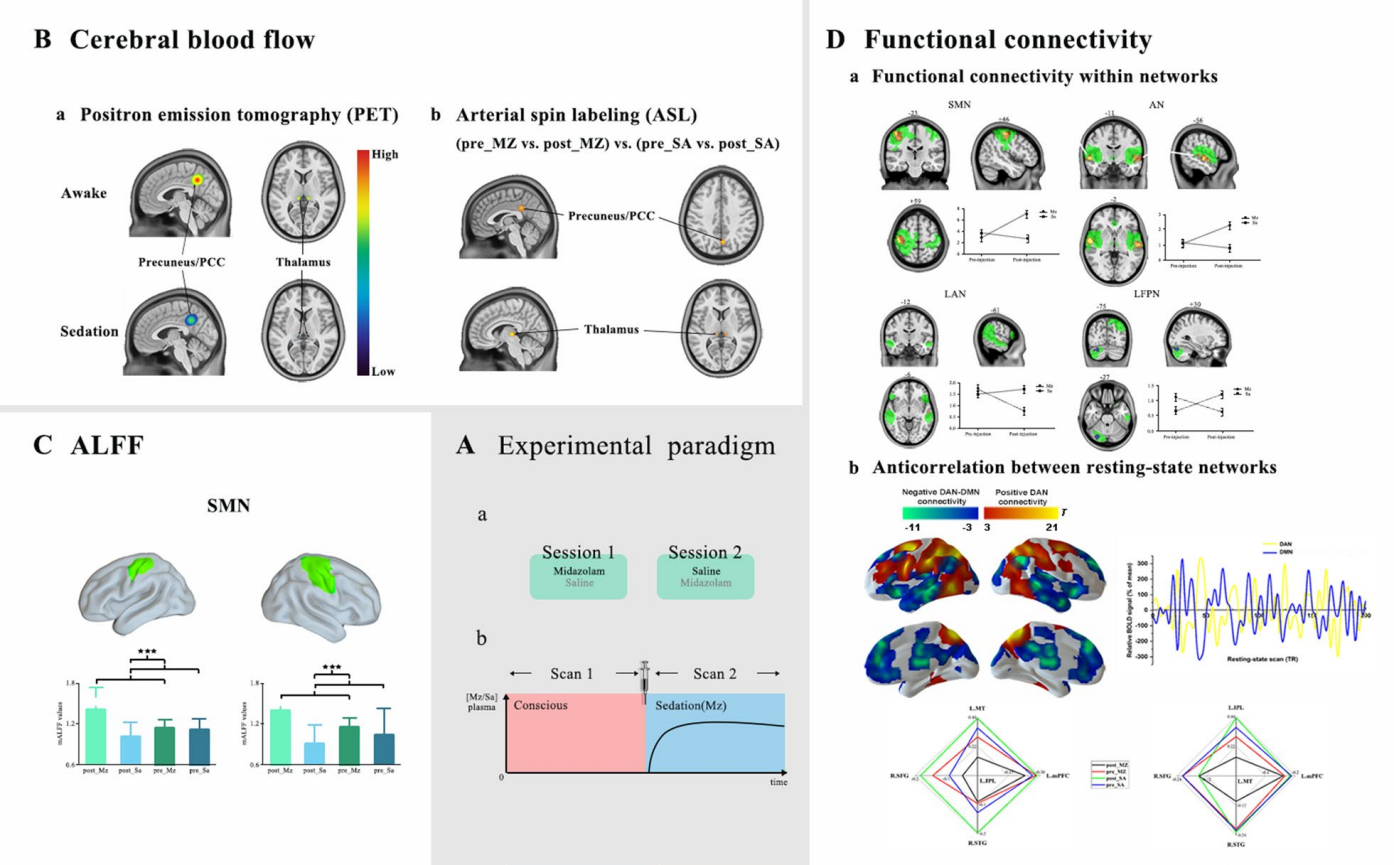
The typical neuroimaging findings associated with midazolam-induced sedation. **A** The experimental paradigm of functional studies with midazolam. (a) Volunteers received midazolam in one session and saline in the other. Two sessions were randomly assigned to either midazolam or saline. (b) Scan 1 represented a pre-injection imaging section and scan 2 represented a post-injection imaging section. **B** Early ASL and PET studies revealed that midazolam-induced sedation was consistently correlated with a reduction in cerebral blood flow in the thalamus and the posterior cortical areas (precuneus or PCC). **C** Significantly increased amplitude of low-frequency fluctuations (ALFF) was observed in lower-level resting-state networks (e.g., SMN) during midazolam-induced sedation. **D** Modified resting-state functional connectivity within and between networks during midazolam-induced sedation. (a) Changes of resting-state functional connectivity within networks during midazolam-induced sedation, adapted from Liang et al. [66]; Wiley, USA. Under sedation, decreased cortico-cortical connectivity was found in higher-order brain networks, including the frontoparietal network (FPN) and language network (LAN). In contrast, functional connectivity in low-level networks was intact, including the sensorimotor network (SMN) and auditory network (AN). (b) Altered resting-state functional connectivity between networks. Midazolam significantly decreased the anticorrelation between the dorsal attention network (DAN) and default mode network (DMN)

On the other hand, positron emission tomography (PET), which enables three-dimensional metabolic and flow studies in the human brain, can also detect changes in the regional cerebral blood flow (rCBF) and has been used to investigate the regions subserving conscious brain activity [40]. A previous study demonstrated that midazolam caused dose-related decreased rCBF in brain regions including the cingulate gyrus, insula, multiple areas in the prefrontal cortex, the thalamus, and parietal and temporal association areas [41]. These findings are consistent with several PET studies that have shown rCBF reduction in both cortical and subcortical brain regions in relation to sedation or anesthesia with various anesthetic agents, including isoflurane, propofol, sevoflurane, and thiopental [42–45]. More importantly, these early PET studies reveal that anesthetic-induced sedation, even to the extent of unconsciousness, is consistently correlated with a reduction in rCBF in the thalamus and the posterior cortical areas (precuneus or PCC) [46]. Decreased rCBF in the frontal and parietal cortices have also been observed during sedation or anesthesia but less consistently than in the thalamus and precuneus/PCC [47]. Overall, these observations suggest that the thalamus and precuneus/PCC are key elements to understanding how anesthetics cause a diminished level of consciousness in humans (Fig. 2B).

## Cortical activity during midazolam-induced sedation

There are several different ways that we could apply to identify the neural substrate of midazolam-induced sedation. One powerful approach is to examine brain regions whose activity changes when subjects who ingested midazolam are asked to perform different types of tasks (e.g., processing words, memory task, and pressing button). Task-related fMRI will help us identify and characterize functionally distinct nodes in the brain and interpret the neural correlates underlying tasks assessing different neural systems which include primary sensory processes (e.g., auditory, motor function) and different cognitive processes (e.g., episodic memory, language processing, and emotion processing) [48].

A number of studies have investigated the effects of midazolam-induced sedation on brain activity during primary sensory stimulation and high-order cognitive processing [49–52]. Most studies have focused on the limits of auditory processing, anticipation to pain and memory because of its sedative, anxiolytic, and anterograde amnesic properties. One fMRI study found that mild sedation with midazolam decreased evoked BOLD responses to auditory stimulus in the auditory cortex [49]. An fMRI study in children suggested that subjects sedated with midazolam exhibited activation in the primary auditory cortex [51]. In another study, parallel results have also been found. During midazolam-induced mild sedation, activation to auditory stimulus was preserved in the superior temporal gyrus, middle temporal gyrus, transverse temporal gyrus and temporal fusiform gyrus [53]. In the deep sedation group, midazolam sedation inhibited activation of the superior temporal gyrus by auditory stimulus [53]. Similarly, recent studies using fMRI and sedation with other anesthetic agents have reported that basic auditory processing remained intact during light sedation and increased depth of sedation abolished reactivity to auditory stimulus [54–56].

However, mild sedation may impair more complex cognitive processing [57]. For example, pain is a subjective experience that involves sensory, affective and cognitive components. Anticipation to pain is an important component of the pain experience which is mediated by a network of brain regions including the secondary somatosensory cortex, the insular regions, the anterior cingulate cortex (ACC), and the thalamus [58–60]. The somatosensory cortex which responses to pain itself was preserved during midazolam-induced mild sedation, whereas the activity associated specifically with anticipation to pain in the ACC, the contralateral anterior insular cortex and the ipsilateral posterior insula was reduced [61]. These findings were interpreted to indicate that basic sensory processing was intact during sedating, while high-order processes, such as pain experience, were eliminated [61]. In addition, in one study, the results also suggested that midazolam-induced mild sedation did not abolish implicit memory but abolished both explicit and implicit memories with deep sedation. The superior temporal gyrus may be key target areas for memory processing affected by deep midazolam sedation [53]. Overall, these studies are consistent with the notion that cortical activity is sequentially impaired from higher-order brain cortices to primary cortical areas in a dose-dependent manner, by midazolam-induced sedation.

## Functional connectivity during midazolam-induced sedation

To date, inherent and spontaneous neural activity of the brain can be well characterized by resting-state functional magnetic resonance imaging (rs-fMRI). It is one of the most important technologies for the non-invasive mapping of brain activity pattern and functional interactions between several large-scale networks known as resting-state networks (RSNs) [62, 63]. It is also widely indicated that low-frequency fluctuations of BOLD fMRI signal in the resting state, an indirect measure of regional brain activity, is correlated with spontaneous neuronal activity [64, 65]. Based on accumulating experimental evidence, midazolam-induced sedation affects within and between network connectivity of the RSNs [66–69]. Some of RSNs are thought of as lower-level networks including the sensorimotor network (SMN), auditory network (AN), and visual network (VN), which relate to sensory processing. For example, the intrinsic signals measured from primary motor cortex, primary sensory cortex, and supplementary motor areas maintain temporally correlated BOLD activity. These regions comprise the SMN, which is involved in processing somatosensory information and planning motor tasks (movement) [70]. Under midazolam-induced sedation, functional connectivity within lower-level networks is preserved, which is consistent with the task fMRI studies reviewed above that have suggested intact activation in primary cortical areas [67]. In recent studies, midazolam-induced light sedation effects on lower-level networks were even elevated [66] (Fig. 2C). The authors interpreted their results as indicating that neural systems autonomously compensate for the drug effect as a direct reaction to the drug injection, which helped to maintain the information processing abilities [66].

By contrast, functional connectivity within high-order networks is significantly disrupted by midazolam-induced light sedation [66]. In general, the high-order networks, including the executive control network (ECN), salience network (SN), frontoparietal network (FPN), dorsal attention network (DAN), language network (LAN), and default mode network (DMN), are responsible for various complex cognitive processing [71, 72]. For example, the ECN is involved in impulse inhibition, ability to respond to an external event, and skills (such as organizing tasks, solving problems) required for goal-directed behavior [73]; the SN is involved in detecting and filtering salient stimuli, conflict monitoring, integrating emotional and sensory stimuli, while simultaneously switching between activating and deactivating the DMN and central executive networks [74]; the FPN assumes a variety of functions, including motor planning and imagery, mental rotation, spatial attention, and coordinating behavior in a rapid, accurate, and flexible goal-driven manner [75]; the DAN is involved in selection of the appropriate response or action necessary for the attention orientation [76]; and the DMN, the most widely studied network, is involved in autobiographic episodic memories, mind wandering, the awareness of self, self-generated and internally directed thought, and self-referential processing [77]. Among them, there is now indisputable evidence that functional connectivity within the ECN, FPN, and LAN is significant decreased during midazolam-induced light sedation [66, 78] (Fig. 2D). For the SN, in our previous work, we only identified a slight reduction in functional connectivity strength by using a lower uncorrected threshold [66]. In line with this, in one recent study, midazolam also showed no significant changes in the functional connectivity strength for the SN [78]. As for the DAN and DMN, results of the drug effect are still inconsistent. In our previous work, no significant functional connectivity changes within the DMN and DAN were observed during midazolam-induced light sedation [66]. In contrast, another study showed that midazolam administration led to a significant reduction in the connectivity strength in the DMN and DAN [78]. Many factors may contribute to these inconsistent results. For example, anesthesia alters functional connectivity in a dose-dependent manner and increasing depth of sedation may abolish functional connectivity within a wide range of RSNs [6, 79]. In addition, the indirect nature of BOLD signal restricts the discrimination of neural from physiological contributions. One recent study assessed the validity of fMRI functional connectivity using simultaneous electroencephalography (EEG)/fMRI in a placebo-controlled design with midazolam. The results suggested that different preprocessing strategies demonstrated the effects on the results of functional connectivity of RSNs [68] (Table 1).

**Table 1:** Neuroimaging studies during midazolam-induced sedation

First author and year	Modality	Sample size	Main findings
Liang et al. 2018 [38]	ASL	*n* = 12 (20–30 years old)	Decreased CBF in the bilateral medial thalamus and precuneus/PCC
Veselis et al. 1997 [41]	PET	*n* = 14 (28.1 ± 5.8 year)	Decreased rCBF in the cingulate gyrus, insula, multiple areas in the prefrontal cortex, the thalamus, and parietal and temporal association areas
Frölich et al. 2017 [49]	fMRI	*n* = 11 (19–40 years old)	Reduction of brain activation in the auditory cortex
Gemma et al. 2009 [51]	fMRI	n = 5 (4,5–6 years old)	Subjects exhibiting activation in the primary auditory cortex
Tian et al. 2010 [53]	fMRI	*n* = 12 (22–38 years old)	Preserved activation in the auditory cortex by auditory stimulus
Wise et al. 2007 [61]	fMRI	*n* = 8 (25 ± 5 year)	Reduced activity in the ACC, the insular cortex
Liang et al. 2015 [66]	fMRI	*n* = 14 (24 ± 3.2 year)	Impaired higher-order cognitive functions prior to lower-level sensory responses
Greicius et al. 2008 [67]	fMRI	*n* = 9 (22–27 years old)	Reduced functional connectivity in the posterior cingulate cortex
Forsyth et al. 2020 [68]	fMRI	*n* = 30 (27.3 ± 6.2 year)	Increased connectivity in sensory networks (SMN, VN), and decreased connectivity in some of the higher cognitive networks (rFPN, pDMN)
Adhikari et al. 2020 [78]	fMRI	*n* = 30 (27.3 ± 6.2 year)	Reduced connectivity in the DMN

*ASL* arterial spin labeling, *CBF* cerebral blood flow, *PCC* posterior cingulate cortex, *PET* positron emission tomography, *rCBF* regional cerebral blood flow, *fMRI* functional magnetic resonance imaging, *ACC* anterior cingulate cortex, *SMN* sensorimotor network, *VN* visual network, *FPN* frontoparietal network, *DMN* default mode network

Recent studies of resting state have revealed that the human brain is intrinsically organized in two brain networks showing a reciprocal pattern of spontaneous activity: an “intrinsic” and an “extrinsic” network [65, 80, 81]. The “intrinsic” network corresponds to the DMN and the “extrinsic” network coincides with the DAN. It is well established that these two systems oppose each other and the anticorrelation between the DAN and DMN seems to serve as an essential neural substrate for flexibly allocating attentional resources, which is important for normal cognitive function [82, 83]. The previous literature suggests that consciousness is typically taken to have two aspects: level and content [84–86]. The content of consciousness refers to awareness or subjective experience [84, 85]. Furthermore, awareness can be divided into two components: awareness of the environment (external) and awareness of self (internal) [87]. External awareness can be defined as the conscious perception of one’s environment through the sensory modalities whereas internal awareness is a mental process that does not require the mediation of external stimuli or sensory input [87]. According to recent literature, activity in the DMN corresponds to internal awareness (i.e., self-related thoughts) and external awareness (i.e., perception of the environment through the senses) correlates to the activity in the DAN [87]. These findings suggest that the anticorrelation between the DAN and the DMN is essential to consciousness. Our recent unpublished work has proved midazolam decreased functional connectivity between the DAN and DMN (Fig. 2D). Consistent with this finding, studies in other anesthesia-induced sedation, sleep and in patients with disorders of consciousness have shown that anticorrelation between the DAN and DMN generally reduces or even disappears [88–91].

In addition to the altered static functional connectivity within and between the RSNs, recent studies of anesthetic-induced sedation or unconsciousness have focused on functional brain dynamics and connectivity patterns [92–95]. One recent study combined graph theory and dynamic functional connectivity to compare resting-state functional MRI data from propofol-anesthetised volunteers and patients with disorders of consciousness, in order to identify consciousness-specific patterns of brain function [94]. The authors demonstrated that loss of consciousness (whether due to propofol anesthesia or traumatic brain injury) is accompanied by reduced functional diversity and integrative capacity in the posterior regions of the brain’ s DMN, especially during temporal states of high integration [94]. Consistent with results of static functional connectivity between the DAN and DMN above, another recent study also compared resting-state functional MRI data from anesthetized volunteers (propofol anesthesia or ketamine anesthesia). The findings demonstrated that the transitions between DMN and DAN were embedded in a “temporal circuit” characterized by a set of trajectories along which dynamic brain activity occurred. Isolation of the DMN and DAN from the temporal circuit was associated with unconsciousness [93].

## Similar and contrasting neuroimaging results from studies with diverse anesthetic agents

In comparison with midazolam-induced sedation, there are other anesthetic agents that have the similar mechanism of action and sites of action for sedation or anesthesia. Like midazolam, propofol is also an intravenous sedative agent with a rapid onset and a short duration of action. It is widely used for initiation and maintenance of anesthesia, combined sedation and regional anesthesia, induction and maintenance of general anesthesia [5]. Propofol produces its hypnotic effects by a positive modulation of the inhibitory function of the neurotransmitter GABA through GABA_A_ receptors like midazolam [96, 97]. Due to similar pathways of anesthetic action, there are numerous similarities between these two drugs of neuroimaging research. First, according to studies focused on propofol-induced changes in rCBF, the drug sharply reduces rCBF in both cortical and subcortical brain regions [40, 45, 98, 99]. In addition to this, an important contribution of the early PET studies is to reveal that propofol-induced anesthesia is consistently associated with a reduction in the medial thalamus, precuneus and the PCC [46]. These findings are highly coincident with the effects of midazolam on the rCBF. These results provide strong evidence that reductions in rCBF in the thalamus and precuneus/PCC is functionally related to decreased level of consciousness independently of nonspecific effects of anesthetic agents. Then, several recent fMRI studies have suggested that propofol decreased cortico-cortical connectivity in higher-order brain networks (e.g., the executive control network) [88, 100–102]. In contrast, functional connectivity in low-level sensory cortices (e.g., sensorimotor network, visual and auditory networks) is preserved, consistent with the studies with midazolam reviewed above [88]. In addition, decreased anticorrelation between the DAN and DMN has been reported during propofol-induced alterations of consciousness [88]. Thus, the abovementioned findings are most likely the common neural correlates of anesthetic-induced alteration of consciousness.

Despite common neural changes related to anesthesia, there are some specific effects on brain function for other anesthetic drugs. Numerous studies have suggested that anesthetic-induced sedation disturbs cerebral connectivity. However, the drug effects on higher-order brain networks still vary among diverse anesthetic agents [6]. For example, in our previous work, no significant functional connectivity changes within the DMN were observed during midazolam-induced light sedation [66]. In contrast, significant reductions in resting-state functional connectivity for DMN were reported during propofol or ketamine-induced mild sedation [78, 88, 89]. Midazolam or propofol-induced decrease in consciousness correlates with decreased functional connectivity within the FPN, whereas ketamine administration leads to no significant reduction in the connectivity strength in the FPN [66, 88, 89]. For the salience network, midazolam-induced sedation barely affects it, whereas the strongest effect of ketamine administration is observed in the salience network [66, 78]. On the other hand, the previous studies are consistent with the notion that auditory network is minimally affected during midazolam or propofol-induced sedation [66, 88]. However, ketamine administration significantly decreases functional connectivity with auditory network [78, 103]. Compared with studies using midazolam, thalamocortical system, which is a bilateral structure and has two thalamic nuclei, the specific and nonspecific divisions, are involved in propofol-induced anesthesia [104–106]. The specific thalamocortical network, which contains dominantly medial and bilateral frontal and temporal areas, is moderately affected, while the nonspecific thalamocortical network, which contains medial frontal and medial parietal areas, is severely suppressed during propofol-induced sedation [104]. These findings have not yet been observed in studies related to midazolam.

## Areas for future research

For decades, anesthetic agents are used to manipulate the global level of consciousness to explore the neural correlates of consciousness in healthy individuals. Combined with various neuroimaging techniques, the studies are consistent with the notion that the cortex and subcortical structures are suppressed sequentially in a dose-dependent manner [6]. Broadly speaking, depending on the anesthetic agent and dose, it may produce different levels and contents of consciousness described as a scale ranging from vivid wakefulness, to light sedation, to deep sedation, final to total unconsciousness [84, 107]. The level of consciousness sometimes represents the degree of arousal which can be assessed by purposeful response to verbal command. The content of consciousness is sometimes used synonymously with awareness which can be determined by cognitive testing [85]. Based on recent literature, the consciousness can be divided into three different states including a state of connected consciousness (awareness of the environment), a state of disconnected consciousness (only awareness of self) and unconsciousness (a complete absence of subjective experience) along with increased anesthetic concentration [85, 108]. Currently, most studies with midazolam still focus on a certain state of consciousness. Coupled with multimodal neuroimaging which combines data obtained from multiple neuroimaging techniques, future work in a larger sample size including participants who have submitted to stepwise increments in anesthetic agent concentration, up to unconsciousness is expected to enable a comprehensive and accurate understanding of neural substrates of consciousness.

There is growing evidence that functional brain dynamics captures important information about cognition and the emergence of momentary neural coalitions forms the basis for complex cognitive functions, such as consciousness [109, 110]. Like many other complex systems, the brain exhibits a wide range of dynamic activity and connectivity patterns that are thought to be fundamental for processing information in the course of cognition [111–113]. However, to date, most of the fMRI research has focused on changes in static functional connectivity patterns during midazolam-induced light sedation [66, 69]. A systematic analysis of changes in temporal and spatial properties of dynamic connectivity networks during midazolam-induced alteration of consciousness is an area for future work. Moreover, functional connectivity describes the statistical dependencies between two or more variables and does not provide information about the directed casual interactions among brain regions [114–116]. Furthermore, functional connectivity analyses make minimal assumptions about the physical mechanisms and may not reflect correlations among neuronal activity. These limitations call for an approach in terms of effective connectivity [114]. Effective connectivity refers causal influence that one neural system exerts over another, either at a synaptic or a population level [114]. The related follow-up studies are expected to identify dynamic effective connectivity changes during midazolam-induced alteration of consciousness for better understanding the detailed circuitry underlying consciousness.

In addition, previous studies suggested that anesthetic agent can indirectly affect BOLD signaling by altering physiological parameters, such as arterial concentration of carbon dioxide. Since individuals are not intubated but spontaneously ventilate during midazolam-induced sedation, respiratory depression associated with intravenous midazolam may have resulted in hypercapnia and increased arterial CO_2_ levels [117]. During this condition, certain regions of gray matter appeared to have greater cerebrovascular responses to changes in the partial pressure of carbon dioxide (PaCO_2_) and partial pressure of oxygen (PaO_2_) than did others [118]. We expect that future work should measure and control both PaCO_2_ and PaO_2_ during MRI procedures that involve BOLD signaling and take into account when interpreting findings.

## Conclusion

In this review, we have summarized neuroimaging mechanisms of midazolam-induced sedation or anesthesia, which is important for understanding how midazolam-induced sedation impacts on various domains of cognition and the patterns of preserved brain activity during sedation. In particular, midazolam-induced alteration of consciousness is specifically associated with a significant decreased CBF in the bilateral medial thalamus and precuneus/PCC. Studies of individuals who are sedated with midazolam suggest that cortical activity is sequentially impaired from higher-order brain cortices to primary cortical areas in a dose-dependent manner. Under midazolam-induced sedation, functional connectivity within lower-level networks is preserved. By contrast, functional connectivity within and between higher-order networks, particularly the anticorrelation between the DAN and the DMN, is seemingly of neuronal origin of consciousness. Nevertheless, although much published work has suggested the effective role of preserved brain activity in the emergence of the conscious awareness, more evidence should still be required to further explore brain mechanisms of midazolam-induced alteration of consciousness.

## Data Availability

This review used available literature relevant to the problem statement from valid databases across the internet.

## Abbreviations

ACC: Anterior cingulate cortex
AN: Auditory network
ASL: Arterial spin labeling
BOLD: Blood oxygen level-dependent
CBF: Cerebral blood flow
DAN: Dorsal attention network
DMN: Default mode network
ECN: Executive control network
EEG: Electroencephalography
fMRI: Functional magnetic resonance imaging
FPN: Frontoparietal network
GABA: Gamma­aminobutyric acid
IV: Intravenous
LAN: Language network
PaCO_2_: Partial pressure of carbon dioxide
PaO_2_: Partial pressure of oxygen
PCC: Posterior cingulate cortex
PET: Positron emission tomography
rCBF: Regional cerebral blood flow
rs-fMRI: Resting-state functional magnetic resonance imaging
RSNs: Resting-state networks
SMN: Sensorimotor network
SN: Salience network
TI: Transitive inference
VN: Visual network
